# Age at Menarche, the Leg Length to Sitting Height Ratio, and Risk of Diabetes in Middle-Aged and Elderly Chinese Men and Women

**DOI:** 10.1371/journal.pone.0030625

**Published:** 2012-03-20

**Authors:** Baqiyyah N. Conway, Xiao-Ou Shu, Xianglan Zhang, Yong-Bing Xiang, Hui Cai, Honglan Li, Gong Yang, Yu-Tang Gao, Wei Zheng

**Affiliations:** 1 Division of Epidemiology, Department of Medicine, Vanderbilt University, Nashville, Tennessee, United States of America; 2 Department of Epidemiology, Shanghai Cancer Institute, Shanghai, People's Republic of China; German Diabetes Center-Leibniz Center for Diabetes Research at Heinrich Heine University Duesseldorf, Germany

## Abstract

**Aims:**

To evaluate the associations of age at menarche and the leg length-to-sitting-height ratio, markers of adolescent growth, with risk of diabetes in later life.

**Materials and Methods:**

Information from 69,385 women and 55,311 men, aged 40–74 years from the Shanghai Women's Health Study and Shanghai Men's Health Study, were included in the current analyses. Diabetes status was ascertained through biennial in person follow-up. Cox models, with age as the time scale, were used.

**Results:**

There were 2369 cases of diabetes (1831 women; 538 men) during an average of 7.3 and 3.6 years of follow-up of the women and men, respectively. In females, menarche age was inversely associated with diabetes risk after adjustment for birth cohort, education, and income (HR = 0.95, 0.92–0.98). In both genders, leg length-to-sitting-height ratio was inversely related to diabetes (HR = 0.88, 0.80–0.97 for men; HR = 0.91, 0.86–0.96 for women) after adjustment for birth cohort, education, and income. Further adjustment for adult BMI at study enrollment completely eliminated the associations of age at menarche (HR = 0.99, 0.96–1.02) and the leg length-to-sitting-height ratio (HR = 1.00, 0.91–1.10 for men; HR = 1.01, 0.96–1.07 for women) with diabetes risk.

**Conclusions:**

Our study suggests that markers of an early age at peak height velocity, i.e. early menarche age and low leg-length-to-sitting height ratio, may be associated with diabetes risk later in life and this association is likely to be mediated through obesity.

## Introduction

Factors associated with early life nutrition have been suggested to be associated with future risk of diabetes and other chronic diseases. Low birth weight, early adiposity rebound [1], early menarche [2], and BMI in late adolescence have all been found to be associated with the future risk of impaired glucose tolerance, the metabolic syndrome, and/or diabetes. Shorter adult stature has been found to be associated with impaired glucose tolerance [3] and gestational diabetes [4]. Few studies have evaluated markers of early life nutrition with diabetes risk in population-based settings. Two previous studies have linked longer leg length and a higher leg-to-trunk length ratio with a lower prevalence of type 2 diabetes [4], [5] and an association between an earlier age at menarche and risk of diabetes later in life [6], [7] has been recently reported.

Diabetes is an increasing problem in developing countries, which have seen increases in height [8], reduction in menarche age, and improvements in economic status [9]. The leg-length-to-sitting-height ratio is considered a marker of prepubertal nutritional status [10] and a marker of the age at pubertal onset [10], with a longer-leg-length to sitting height ratio associated with a later age at pubertal onset [10], age at menarche [11], and peak height velocity [12]. We evaluated the relationship of markers of adolescent growth with risk of diabetes in later life using data from the Shanghai Women's Health Study and the Shanghai Men's Health Study, two large population-based cohort studies of approximately 130,000 middle-aged and elderly women and men.

## Methods

The Shanghai Women's Health Study and the Shanghai Men's Health Study are population-based prospective studies of Chinese women aged 40 to 70 years and men aged 40 to 74 years living in urban communities of Shanghai. The two cohort studies were designed to investigate the association of diet and lifestyle factors with chronic diseases. The study design, methods, and baseline questionnaires have been previously described [13], [14]. Briefly, trained personnel recruited 74,942 women between March 1997 and May 2000 and 61,499 men between March 2002 and June 2006 and conducted in-person interviews. The overall response rates were 92% for women and 75% for men. The study protocols were approved by the institutional review boards of all institutes involved in the studies, and written informed consent was obtained from all participants prior to interview. During the interview, data on dietary habits, physical activity, reproductive history, educational attainment, income, occupation, and physician diagnosis of specific chronic diseases, including diabetes, were collected using structured questionnaires. The cohorts are followed-up by biennial in-person survey and record linkage with the Shanghai vital statistic registry. During follow-up, each participant was asked whether he/she had been diagnosed by a physician to have diabetes since study enrollment. Participants who reported a physician diagnosis of diabetes were further asked if they received positive results on blood glucose tests for having a blood glucose concentration ≥11.1 mmol/L, or on two separate occasions a fasting glucose concentration of ≥7 mmol/L. Diabetes cases included in this study met at least one of the following criteria: a fasting glucose concentration of ≥7 mmol/L on two separate occasions, ≥11.1 mmol/L glucose concentration on an oral glucose tolerance test and/or use of a hypoglycemic agent. All other self-reported cases of diabetes were excluded from analyses.

Anthropometric data was collected during the baseline interview. Standing and sitting height, and weight at baseline were measured by the trained interviewers according to a standardized protocol. Body mass index (BMI) was calculated as the weight in kilograms divided by the square of the height in meters. Information on age at menarche was collected on the baseline questionnaire.

We categorized birth cohorts into eight categories by approximately three-year intervals. These categories were chosen to coincide with major political and societal changes that have resulted in a major impact on food and nutrient availability to the general Chinese population in China. For example, the 1946–1949 birth cohort was born during the Chinese civil war. The major famine in Chinese recent history occurred between 1959 and 1961 [15]. This is the period when the cohort of 1946–1949 reached their adolescence. The famine may have temporarily delayed menarche age among pre-puberty girls and had an impact on leg/standing height ratio, as has been observed in Europe [16]. There were 7356 (3492 women; 3864 men) participants who reported having diabetes at baseline and another 1,805 tested positive for glucose in their urine at baseline, and these participants were excluded from analyses. Because the association between height components with age at puberty onset has been shown to vary by sex [10], analyses were conducted sex-specifically. General linear modeling analysis was used to determine the age-adjusted differences in continuous data. The chi-square test was used to determine differences in categorical data. Cox proportional hazards analysis with age as the time scale was used to determine the multivariable adjusted association of age at menarche and the leg-length-to-sitting-height ratio with risk of diabetes. Covariates adjusted in the analysis include birth cohort (modeled categorically), educational attainment level, and household income. Additional analyses with further adjustment for BMI at study enrollment were carried out to examine whether BMI was the mediator for early life exposure associated with subsequent risk of diabetes in later life. Finally, further adjustment BMI at age 20, physical activity during adolescence (participation in a team sport), and smoking prior to age at menarche (for analyses with age at menarche) or prior to age 20 was made. As the correlation between BMI at age 20 and baseline BMI was modest (r = 0.23, p<0.0001) both BMI at age 20 and BMI at baseline were included in the same model (using the residuals of BMI at study baseline regressed on BMI at age 20 resulted in identical estimates). All analyses were conducted using SAS 9.1.3 (Cary, N.C.).

## Results

Participants were followed for a mean of 7.3 (±1.3) years (women) and 3.6 (1.3) years (men). Characteristics of study participants by diabetes status are presented in Table 1. Participants who developed diabetes (1831 women and 538 men) were older, less educated, and in women, from a lower income household. Birth weight did not differ between cases and noncases (data not shown). Baseline BMI (at study enrollment) was significantly higher in those who developed diabetes compared to those who did not.

**Table 1:** Age-adjusted Characteristics of Study Participants by Incident Diabetes Status: the Shanghai Women's and Shanghai Men's Health Study, mean (SD) or % (n).

	Women		Men	
	Cases (n = 1831)	Non-cases (n = 67,554)	Cases (n = 538)	Non-cases (n = 54,773)
**Age at interview (years)**	56.0 (8.9)	51.5 (8.9)*	57.0 (9.6)	54.4 (9.6)*
**Age at diagnosis (years)**	60.1 (2.0)	N/A	58.5 (0.91)	N/A
**Education**				
***Less than middle school***	35.1 (642)	19.2 (12,992)*	8.1 (43)	6.2 (3,320)*
***Middle school or high school***	54.3 (994)	66.7 (45,043)	69.5 (371)	70.1 (37,865)
***College and above***	10.7 (195)	14.1 (9,507)	22.5 (120)	23.7 (12,809)
**Income** **				
***Low***	32.9 (603)	26.8 (18,106)*	52.7 (283)	55.0 (30,053)
***Middle***	38.7 (709)	38.8 (26,207)	37.4 (201)	35.2 (19,243)
***High***	28.4 (519)	34.4 (23,226)	9.9 (53)	9.8 (5,360)
**BMI at age 20 (kg/m^2^)**	19.4 (2.7)	19.6 (2.6)*	19.7 (2.1)	19.5 (2.1)
**Baseline BMI (kg/m^2^)**	26.4 (3.3)	23.8 (3.3)*	26.0 (3.1)	23.6 (3.1)*
**Started smoking before age 20 years** **	0.27 (5)	0.23 (154)	15.4 (83)	18.6 (10,212)
**Participated in a team sport in adolescence** **	11.4 (209)	12.5 (8,454)	22.1 (119)	22.4 (12,261)

N/A = not applicable

* = p<0·05

**Low income = 1: less 1000 yuans, Middle income = 2: 1000–1999 yuans, High income = 3: > = 2000 yuans.

Univariate analysis suggested that a later age at menarche was related to a reduced risk of diabetes. Compared to the lowest quintile, higher quintiles of age at menarche were associated with a reduced risk of diabetes (Table 2). After adjustment for birth cohort, education, household income (markers of childhood and current socioeconomic status) (Model 2), a statistically significant inverse association was observed which disappeared after further adjustment for baseline BMI (Model 3). Additional adjustment for BMI at age 20 and participation in a team sport during adolescence (Model 4) had no additional effect beyond that observed in Model 3, although a lower BMI at age 20 and a higher baseline BMI were associated with a higher risk of diabetes (data not shown).

**Table 2:** Risk of Diabetes in Middle Age by Age at Menarche, HR (95% CI).

	Model 1	Model 2	Model 3	Model 4
**Quintiles of Age at menarche**				
**Quintile 1**	1.00	1.00	1.00	1.00
**Quintile 2**	0.87 (0.75–1.02)	0.84 (0.72–0.99)	0.91 (0.78–1.07)	90 (0.77–1.05)
**Quintile 3**	0.93 (0.80–1.08)	0.86 (0.74–1.01)	0.96 (0.83–1.12)	0.95 (0.81–1.11)
**Quintile 4**	0.92 (0.79–1.08)	0.83 (0.70–0.97)	0.98 (0.83–1.16)	0.95 (0.81–1.12)
**Quintile 5**	0.87 (0.74–1.02)	0.74 (0.63–0.88)	0.91 (0.77–1.08)	0.88 (0.75–1.05)
**Continuous** *	0.98 (0.95–1.01)	0.95 (0·92–0·98)	0.99 (0.96–1.02)	0.98 (0.95–1.01)

**Descriptive statistics for age at menarche by quintiles of age at menarche are: Quintile 1: mean = 12 years, range = 8–13 years, std = 0.56 years; Quintile 2: mean = 14years, range = 14–14 years, std = 0 years; Quintile 3: mean = 15 years, range = 15–15 years, std = 0 years; Quintile 4: mean = 16 years, range = 16–16 years, std = 0 years; Quintile 5: mean = 17 years, range = 17–26 years, std = 0. 89 years.**

*Expressed as per standard deviation change.

Model 1: univariate analyses. Model 2 controlled for birth cohort, education and income. Model 3 controlled for birth cohort, education, income, and BMI at baseline. Model 4 controlled for birth cohort, education, income, BMI at age 20, BMI at baseline, and participation in team sports during adolescence.

Table 3 presents the relative risk of diabetes by the leg-length-to-sitting-height ratio. In both men and women, the leg-length-to-sitting-height ratio was inversely associated with risk of diabetes. This inverse association persisted in multivariate analysis with adjustment for birth cohort, education and income. When evaluated as a continuous variable, each 1 standard deviation increase in the leg length-to-sitting-height ratio was associated with a 9% reduction in risk of diabetes in women and a 12% risk reduction in men (Model 2). After further controlling for baseline BMI, the leg-length-to-sitting-height ratio was no longer associated with risk of diabetes in either women or men (Model 3). Further adjustment for smoking prior to age 20, BMI at age 20, baseline BMI, participation in team sports during adolescence, and smoking prior to age 20 (Model 4) had no additional effect beyond that of adjustment for baseline BMI in Model 3.

**Table 3:** Risk of Diabetes in Middle-aged and Older Chinese Women and Men by the Leg–Length-to-Sitting-Height Ratio, HR (95% CI).

	Model 1	Model 2	Model 3	Model 4
***Women***				
**Quintiles of Leg-Length-to-Sitting-Height Ratio**				
**Quintile 1**	1.00	1.00	1.00	1.00
**Quintile 2**	0.98 (0.84–1.15)	0.99 (0.84–1.15)	1.13 (0.97–1.32)	1.12 (0.96–1.31)
**Quintile 3**	0.87 (0.74–1.02)	0.87 (0.74–1.03)	1.07 (0.91–1.26)	1.06 (0.90–1.25)
**Quintile 4**	0.94 (0.80–1.10)	0.94 (0.80–1.10)	1.21 (1.03–1.42)	1.19 (1.01–1.39)
**Quintile 5**	0.77 (0.65–0.91)	0.77 (0.66–0.91)	1.06 (0.90–1.26)	1.03 (0.87–1.22)
**Continuous** *	0.90 (0.85–0.95)	0.91 (0.86–0.96)	1.01 (0.96–1.07)	1.00 (0.95–1.06)
***Men***				
**Quintiles of Leg-Length-to-Sitting-Height Ratio**				
**Quintile 1**	1.00	1.00	1.00	1.00
**Quintile 2**	0.65 (0.49–0.88)	0.65 (0.48–0.88)	0.76 (0.56–1.03)	0.76 (0.56–1.03)
**Quintile 3**	0.66 (0.49–0.89)	0.68 (0.51–0.92)	0.85 (0.63–1.15)	0.85 (0.63–1.15)
**Quintile 4**	0.80 (0.61–1.07)	0.84 (0.63–1.11)	1.10 (0.82–1.47)	1.09 (0.82–1.46)
**Quintile 5**	0.66 (0.49–0.89)	0.66 (0.50–0.89)	0.93 (0.69–1.25)	0.92 (0.68–1.24)
**Continuous** *	0.88 (0.79–0.97)	0.88 (0.80–0.97)	1.00 (0.91–1.10)	0.99 (0.90–1.09)

**Descriptive statistics for the leg-length-to-sitting-height ratio by quintiles of the leg-length-to-sitting-height in women are as follows: Quintile 1: mean = 0.80, range = 0.43–0.82, std = 0.02; Quintile 2: mean = 0.84, range = 0.82–0.85, std = 0.008; Quintile 3: mean = 0.86, range = 0.85–0.88, std = 0.007; Quintile 4: mean = 0.89, range = 0.88–0.91, std = 0.009; Quintile 5: mean = 0.95, range = 0.91–2.02, std = 0.05.**

**Descriptive statistics for the leg-length-to-sitting-height ratio by quintiles of the leg-length-to-sitting-height in men are as follows: Quintile 1: mean = 0.82, range = 0.43–0.84, std = 0.02; Quintile 2: mean = 0.85, range = 0.84–0.87, std = 0.007; Quintile 3: mean = 0.88, range = 0.87–0.89, std = 0.006; Quintile 4: mean = 0.90, range = 0.89–0.91, std = 0.008; Quintile 5: mean = 0.94, range = 0.91–1.46, std = 0.03.**

*Expressed as per standard deviation change.

Model 1: univariate analyses. Model 2 controlled for birth cohort, education, and income. Model 3 controlled for birth cohort, education, income, and BMI at baseline. Model 4 controlled for birth cohort, education, income, smoking before age 20, BMI at age 20, BMI at baseline, and participation in team sports during adolescence.

The risk of diabetes associated with total height and the individual height components are presented in Tables 4 and 5 for women and men, respectively. In women, there was a positive association with total height that emerged after controlling for study baseline BMI, but that disappeared upon further adjustment for BMI at age 20, smoking prior to age 20, and participation in team sports during adolescence. There was also a positive association with sitting height in women that disappeared after adjustment for baseline BMI (models 3 and 4), but an inverse association with leg length that disappeared after multivariable adjustment in women. In men, there was no association observed for total height or sitting height, but an inverse association was observed for leg length that disappeared upon multivariable analysis.

**Table 4:** Risk of Diabetes in Middle-aged and Older Chinese Women by Height Components, HR (95% CI).

	Quintile 1	Quintile 2	Quintile 3	Quintile 4	Quintile 5	Continuous*
**Total Height**						
**Model 1**	1.00	1.09 (0.93–1.27)	0.98 (0.84–1.15)	1.09 (0.93–1.28)	1.02 (0.86–1.21)	1.00 (0.94–1.05)
**Model 2**	1.00	1.12 (0.96–1.30)	1.03 (0.88–1.21)	1.16 (0.98–1.36)	1.11 (0.93–1.32)	1.03 (0.97–1.09)
**Model 3**	1.00	1.19 (1.02–1.39)	1.14 (0.97–1.33)	1.26 (1.07–1.48)	1.25 (1.05–1.49)	1.07 (1.02–1.13)
**Model 4**	1.00	1.17 (1.00–1.36)	1.11 (0.94–1.30)	1.21 (1.03–1.43)	1.17 (0.98–1.39)	1.05 (0.99–1.11)
**Leg Length**						
**Model 1**	1.00	0.96 (0.82–1.11)	0.92 (0.79–1.07)	0.94 (0.80–1.10)	0.88 (0.74–1.03)	0.94 (0.89–0.97)
**Model 2**	1.00	0.97 (0.84–1.14)	0.94 (0.81–1.10)	0.98 (0.83–1.15)	0.93 (0.79–1.10)	0.96 (0.91–1.01)
**Model 3**	1.00	1.09 (0.93–1.27)	1.11 (0.95–1.30)	1.20 (1.02–1.41)	1.20 (1.01–1.42)	1.05 (1.00–1.11)
**Model 4**	1.00	1.06 (0.91–1.24)	1.09 (0.93–1.27)	1.16 (0.99–1.36)	1.12 (0.94–1.33)	1.03 (0.98–1.09)
**Sitting Height**						
**Model 1**	1.00	1.13 (0.98–1.32)	1.05 (0.89–1.25)	1.12 (0.95–1.31)	1.27 (1.08–1.50)	1.08 (1.02–1.14)
**Model 2**	1.00	1.17 (1.01–1.36)	1.10 (0.92–1.31)	1.17 (1.00–1.38)	1.36 (1.15–1.61)	1.11 (1.05–1.17)
**Model 3**	1.00	1.13 (0.97–1.31)	1.01 (0.85–1.20)	1.10 (0.93–1.29)	1.19 (1.00–1.40)	1.05 (1.00–1.11)
**Model 4**	1.00	1.11 (0.96–1.29)	0.98 (0.82–1.16)	1.06 (0.90–1.25)	1.14 (0.96–1.35)	1.04 (0.98–1.10)

**Descriptive statistics for height components by quintiles in women**: **Height (m):** Quintile 1: mean = 1.50, range = 1.19–1.54, std = 0.03; Quintile 2: mean = 1.55, range = 1.54–1.57, std = 0.008; Quintile 3: mean = 1.58, range = 1.57–1.60, std = 0.008; Quintile 4: mean = 1.61, range = 1.60–1.63, std = 0.009; Quintile 5: mean = 1.66, range = 1.63–1.86, std = 0.02. **Leg Length (cm):** Quintile 1: mean = 68.3, range = 0.40–0.70, std = 1.86; Quintile 2: mean = 71.4, range = 70–72.3, std = 0.59; Quintile 3: mean = 73.4, range = 72.3–74.0, std = 0.54; Quintile 4: mean = 75.4, range = 74.05–76.5, std = 0.60; Quintile 5: mean = 78.9, range = 76.5–105.0, std = 2.18. **Sitting height (cm):** Quintile 1: mean = 80.3, range = 52.0–82.0, std = 2.13; Quintile 2: mean = 83.5, range = 82.1–84.0, std = 0.53; Quintile 3: mean = 85.0, range = 84.1–85.9, std = 0.30; Quintile 4: mean = 86.5, range = 86.0–87.0, std = 0.47; Quintile 5: mean = 89.2, range = 87.1–105.0, std = 1.48.

*Expressed as per standard deviation change. Model 1: univariate analyses. Model 2 controlled for birth cohort, education, and income. Model 3 controlled for birth cohort, education, income, and BMI at baseline. Model 4 controlled for birth cohort, education, income, smoking before age 20, BMI at age 20, BMI at baseline, and participation in team sports during adolescence.

**Table 5:** Risk of Diabetes in Middle-aged and Older Chinese Men by Height Components, HR (95% CI).

	Quintile 1	Quintile 2	Quintile 3	Quintile 4	Quintile 5	Continuous*
**Height**						
**Model 1**	1.00	0.88 (0.65–1.19)	0.93 (0.70–1.24)	0.84 (0.61–1.16)	0.92 (0.68–1.25)	0.97 (0.87–1.07)
**Model 2**	1.00	0.87 (0.64–1.19)	0.96 (0.71–1.28)	0.88 (0.64–1.21)	0.97 (0.72–1.32)	0.99 (0.90–1.10)
**Model 3**	1.00	0.93 (0.68–1.26)	1.00 (0.75–1.34)	0.94 (0.69–1.29)	1.02 (0.75–1.39)	1.01 (0.91–1.12)
**Model 4**	1.00	0.92 (0.68–1.25)	0.97 (0.74–1.32)	0.92 (0.67–1.27)	0.99 (0.73–1.36)	1.00 (0.90–1.11)
**Leg Length**						
**Model 1**	1.00	1.02 (0.77–1.36)	1.07 (0.80–1.43)	0.87 (0.64–1.18)	0.74 (0.53–1.02)	0.90 (0.82–0.99)
**Model 2**	1.00	1.01 (0.76–1.36)	1.10 (0.82–1.47)	0.90 (0.66–1.23)	0.77 (0.56–1.06)	0.92 (0.83–1.01)
**Model 3**	1.00	1.14 (0.85–1.53)	1.29 (0.96–1.73)	1.10 (0.81–1.50)	0.97 (0.70–1.34)	1.00 (0.91–1.10)
**Model 4**	1.00	1.13 (0.84–1.52)	1.28 (0.95–1.71)	1.08 (0.79–1.48)	0.94 (0.68–1.31)	1.00 (0.90–1.10)
**Sitting Height**						
**Model 1**	1.00	0.77 (0.55–1.07)	1.05 (0.78–1.41)	0.98 (0.71–1.36)	1.19 (0.87–1.64)	1.07 (0.97–1.19)
**Model 2**	1.00	0.78 (0.56–1.09)	1.10 (0.81–1.48)	1.01 (0.73–1.41)	1.25 (0.91–1.72)	1.10 (0.99–1.22)
**Model 3**	1.00	0.74 (0.53–1.04)	0.98 (0.73–1.32)	0.88 (0.63–1.22)	1.00 (0.72–1.38)	1.02 (0.92–1.12)
**Model 4**	1.00	0.74 (0.53–1.03)	0.97 (0.72–1.31)	0.87 (0.62–1.21)	0.98 (0.71–1.35)	1.01 (0.91–1.12)

**Descriptive statistics for height components by quintiles in men:** **Height (m):** Quintile 1: mean = 1.62, range = 1.15–1.65, std = 0.03; Quintile 2: mean = 1.67, range = 1.65–1.69, std = 0.009; Quintile 3: mean = 1.70, range = 1.69–1.72, std = 0.008; Quintile 4: mean = 1.73, range = 1.72–1.75, std = 0.008; Quintile 5: mean = 1.78, range = 1.75–1.96, std = 0.03. **Leg Length (cm):** Quintile 1: mean = 74.1, range = 37–76, std = 2.02; Quintile 2: mean = 77.5, range = 76.1–78.9, std = 0.61; Quintile 3: mean = 79.5, range = 79–80, std = 0.47; Quintile 4: mean = 81.4, range = 80–82.5, std = 0.61; Quintile 5: mean = 84.9, range = 82.5–101, std = 2.06. **Sitting height (cm):** Quintile 1: mean = 85.6, range = 56.087.9, std = 1.84; Quintile 2: mean = 88.6, range = 88.0–90.0, std = 0.53; Quintile 3: mean = 90.5, range = 90.0–91.5, std = 0.53; Quintile 4: mean = 92.4, range = 91.6–93.1, std = 0.47; Quintile 5: mean = 95.2, range = 93.1–108.0, std = 1.51.

*Expressed as per standard deviation change. Model 1: univariate analyses. Model 2 controlled for birth cohort, education, and income. Model 3 controlled for birth cohort, education, income, and BMI at baseline. Model 4 controlled for birth cohort, education, income, smoking before age 20, BMI at age 20, BMI at baseline, and participation in team sports during adolescence.

## Discussion

Few studies have evaluated markers of growth during adolescence and diabetes risk after age 40. In this report we have shown that factors suggestive of a later age at peak height velocity, i.e. a greater leg-length-to-sitting-height ratio, and in women, a later age at menarche, are associated with a reduced risk of diabetes in later life. These associations were completely attenuated after adjustment for recent BMI, suggesting obesity mediates some of this risk. Other mechanisms by which a later age at pubertal growth acceleration and a later age at menarche may be related to the risk of diabetes in adulthood include poor in utero and early childhood nutrition and hormonal factors involved in growth and puberty regulation.

The leg-length-to-sitting-height ratio is a marker of timing of puberty and peak height velocity [17]. Prior to the pubertal growth spurt, growth is more rapid in the legs, whereas during and after the pubertal growth spurt, growth is more rapid in the trunk and head. Thus the leg length to sitting height ratio progressively rises until around the age of peak height velocity in both males and females and then begins to decline [18]. An earlier age at peak height velocity, suggestive of an earlier entry into puberty, and subsequent fusion of the epiphyseal growth plate, would result in an earlier cessation of long bone growth, but not truncal growth, and thus a lower adult leg-length-to-sitting-height ratio. To our knowledge, only a few studies have evaluated the leg length, or leg length to sitting height ratio, in association with risk of type 2 diabetes. Shorter adult stature, a shorter leg length, and a shorter leg-length-to-standing-height ratio have been linked to the prevalence of type 2 diabetes in the National Health and Nutrition Examination Survey (NANES) III [4]. Although height was no longer statistically significant in that population after further adjustment for potential confounders such as a parental history of diabetes and socioeconomic status, leg length and the leg-length-to-standing-height ratio remained significantly associated with the prevalence of diabetes [4]. Upon further adjustment for body fat, the leg-length-to-standing-height ratio remained associated with the prevalence of type 2 diabetes.

The onset of puberty requires reaching a critical level of either height or body fat. Poor *in utero* nutrition leading to insulin resistance [19], [20] in childhood and thus increased insulin levels may result in increased anabolism in bone, muscle, and adipose tissue and an earlier age at reaching these critical levels of height and/or body fat. In females, hyperinsulinemia is associated with both accelerated onset and progression through puberty [21].

Most previous studies, while finding an association between age at menarche and glucose tolerance [2], [22], [23], have failed to find an association between age at menarche and risk of type 2 diabetes [2], [24], possibly, as suggested by Lakshman et al, due to low statistical power [7]. In a study of 13,308 female participants of the Norfolk cohort of the European Prospective Investigation into Cancer and Nutrition (EPIC-Norfolk), Lakshman et al found that an earlier age at menarche was associated with the risk of type 2 diabetes [7]. However, this association was accounted for by body mass index in adulthood. In our study as well, BMI partially mediated the association between age at menarche and diabetes risk.

The effect of both age at menarche and final attained height on risk of diabetes may be mediated through estrogen. Estrogen is responsible for the onset of menstruation and the acceleration of growth during puberty. However, estrogen has a biphasic effect on linear growth [25]. At relatively low levels it causes an increase in growth velocity, while at high levels it is responsible for the closure of the epiphyseal plates, resulting in the cessation of long bone growth and thus the end of linear growth in adolescence. A later age at menarche has been repeatedly shown to be related to increased adult height [26]. In our population as well, a later age at menarche was associated with an increased final attained height (data not depicted). Endogenous levels of estradiol are positively associated with insulin and glucose and with type 2 diabetes [27], although exogenous estrogen replacement has been shown to increase insulin sensitivity and decrease the risk of type 2 diabetes. The latter has been suggested to be dependent on the route of estrogen delivery [28], [29]. Transdermal estradiol therapy was shown to increase plasma glucose levels in women [30], while oral estrogen therapy decreases glucose levels [29].

Estrogen also inhibits production of insulin-like growth factor-1 (IGF-1) [31], [32] and low levels of IGF-1 are associated with an increased risk type 2 diabetes [33]. IGF-1 is a hormone with structural and functional homology to insulin and helps regulate postnatal somatic and bone growth [34]. Serum levels of IGF-1 follow the pattern of somatic growth, increasing in puberty, and declining down toward prepubertal levels after adolescence [35]. IGF-1 also promotes pancreatic growth, increases in beta cell mass, and protects against islet cell apoptosis [36]. Although postnatal pancreatic growth is greatest in the first year of life, the pancreas does continue to grow during childhood and adolescence. If pancreatic growth follows a pattern similar to the rise of IGF-1 with somatic growth, it is possible that earlier cessation of linear growth, as suggested by an earlier age at peak height velocity, would result in a reduced final pancreatic size and beta cell mass as it does with a reduced final attained height. In intrauterine growth deficiency resulting in decreased pancreatic mass, decreased glucose tolerance is observed [37].

As higher prepubertal BMI is associated with an earlier entry into puberty and an earlier age at menarche, and being overweight in childhood and adolescence tends to track into adulthood, it is possible that our markers of somatic growth are just predicting adult BMI. Although we did not have data on BMI before age 20 and thus could not adjust for prepubertal BMI, overweight or obesity during adolescence appeared to be very low in our study population as the mean BMI at age 20 was 19.6 and 19.5 kg/m^2^ in both women and men, respectively; only 2% of the women 1% of the men had a BMI above 25 kg/m^2^ at age 20. Adjustment for BMI at age 20 had no additional affect beyond that of BMI in older adulthood, i.e. BMI at baseline (enrollment). Adjustment for baseline BMI accounted for the association of age at menarche and the leg-length-to-sitting height ratio with diabetes incidence in middle-age and beyond.

Differences in height and height components are determined by genetic influences in addition to early life nutritional exposure [38], and thus the generally weaker to null associations between leg length and total height with diabetes in our study are not unexpected. By using the leg-length-to-sitting-height ratio in this racially homogenous population, we were able to minimize the genetic influence on height. We do not have a good explanation for the positive association between sitting height and diabetes incidence in women; however, a positive association between sitting height and insulin resistance has been observed by others [38], [39]. It has been shown that in Chinese children leg length growth occurs more rapidly prior to puberty, while truncal growth occurs more rapidly during puberty [18]. The epiphyseal plates of the trunk are the last to close and thus truncal bones are the last to stop growing [40]; nevertheless total final attained height is more largely determined by long bone, particularly leg, growth. Data from the Guangzhou Biobank Cohort Study showed that an earlier age at puberty was associated with shorter legs, a taller sitting height, and a shorter leg-length-to-sitting-height ratio in women, but was significantly associated only with a taller sitting height in men [10]. They also showed that the impact of puberty on leg length was greater in females than in males [10]. A caveat needs to be put forth in drawing in conclusions about sex differences in the relationship with puberty onset with diabetes risk based on the results of this study, as the small sex difference in our results may be due to the shorter follow-up time and smaller number of incident diabetes cases in males than in females.

Strengths of our study include the large sample size, the high response rates, measured standing and sitting height and BMI, and a population-based study design. We also controlled for smoking prior to age 20 as a large percentage of our males were smoking during adolescence and smoking during adolescence has been shown to reduce linear growth, particularly in boys [41]. A potential limitation of our study was that age of menarche was assessed by recall approximately forty years later. However, it has been shown that actual age at menarche was strongly correlated with age at menarche recalled thirty years later by 407 participants in the Newton Girls Study (r = 0.79) [42] with a mean difference of 0.08 years. Another study of 50 year-old women showed a moderately high correlation (r = 0.67). Any influence on our results due to error in recall of age at menarche is likely to be nondifferential which would result in underestimation of the association under study. We did not have data on BMI during early childhood or prior to menarche and thus our data cannot determine whether it was early childhood over-nutrition, nutritional deprivation, or early pubarche unrelated to nutritional status that was driving the results observed between a lower age at menarche or a shorter leg-length to sitting height ratio and a higher risk of diabetes later in life. Another limitation was that we did not have measured data on growth during adolescence or the leg-length-to-sitting-height ratio in young adulthood, nor did we have data on IGF-1, estrogen, or pancreatic size, limiting our discussion of these mechanisms to speculation. Finally, the much smaller number of cases in men, mainly due to the shorter follow-up time, limited our ability to evaluate the potential differences that may exist between men in women in the association of height components with diabetes incidence. In conclusion, we found that markers suggestive of an early age at onset of puberty and peak somatic growth were related to an increased diabetes risk in late adulthood among both men and women. As *in utero* and early childhood nutrition are known to affect somatic growth, our study suggests that care should be given to prenatal and postnatal/early childhood nutrition for prevention of diabetes in adulthood. Finally, our data provide further evidence that weight management in later adulthood may be protective against diabetes, particularly in those who demonstrated an early age at menarche and linear growth maturation.
